# Facile and Eco-Friendly Fabrication of Colored and Bioactive Silk Materials Using Silver Nanoparticles Synthesized by Two Flavonoids

**DOI:** 10.3390/polym10040404

**Published:** 2018-04-04

**Authors:** Yuyang Zhou, Ren-Cheng Tang

**Affiliations:** National Engineering Laboratory for Modern Silk, College of Textile and Clothing Engineering, Soochow University, 199 Renai Road, Suzhou 215123, China; ldxyzyy@163.com

**Keywords:** silk fiber, silver nanoparticles, biosynthesis, flavonoids, antibacterial activity, antioxidant activity

## Abstract

Recently, there has been an increasing tendency towards the functionalization of silk using silver nanoparticles (AgNPs) to inhibit bacterial multiplication and disease spread. Considering environmental factors and sustainable development, the preparation of AgNPs using natural extracts is becoming a research hotspot. This study aims at fabricating colored and bioactive silk fabric using AgNPs synthesized by two representative flavonoids (quercetin and rutin). The effects of pH, temperature, and flavonoid concentration on the yield and particle size of AgNPs were studied. The color features and functionalities of the AgNPs-treated silk were also evaluated. The results showed that the AgNPs synthesized by quercetin were generated faster but displayed poorer size uniformity than those prepared by rutin. The as-prepared AgNPs showed good stability. The AgNPs prepared by rutin displayed a more uniform distribution on silk than those synthesized by quercetin. The antibacterial activity of AgNPs-treated silk remained over 90% against *E. coli* and *S. aureus* even after 30 washing cycles. The antioxidant activity of the treated silk gradually decreased during washing. The present research proposes a facile and eco-friendly method for the preparation of AgNPs-coated silk material using flavonoids, which can serve as hygiene-related and medical textile materials.

## 1. Introduction

Silk, a natural and protein-based biopolymer generated by *Bombyxmori* silkworms, has enjoyed a great reputation as a top-grade textile material for thousands of years due to its smooth texture, elegant appearance, and wearing comfort [1]. In recent years, silk has also been proved to exhibit good biocompatibility, non-toxicity and non-irritancy, and superior mechanical properties including strength and extensibility as well as toughness, which is considered to be an excellent biomaterial for medical application [1,2,3]. Silk textile materials, due to their constant contact with human skin and large surface area, high protein content, and moisture retaining ability, can create suitable circumstances for bacterial growth and multiplication, leading to a range of adverse impacts on textile substrate and consumers [4]. For medical applications, silk materials should be non-allergenic, non-toxic, antibacterial, and anti-inflammatory in order to meet strict requirements and high standards [2,3]. Thus, the applications of silk materials are hindered to a large extent.

Silver nanoparticles (AgNPs), which are famous for broad-spectrum and high-efficient antibacterial effects due to their unique properties (e.g., nano-scale size, various shapes, and large surface area), have been widely used in many fields such as electronics, cosmetics, coatings, packaging, and biotechnology [5,6,7]. The synthesis and applications of AgNPs have already become a research hotspot in recent years [8,9]. A variety of techniques including laser ablation, gamma irradiation, electron irradiation, chemical reduction, photochemical methods, biological synthetic methods, etc. have been applied in AgNPs synthesis [10,11,12]. However, most of the conventional syntheses require the use of hazardous chemicals and generate toxic organic byproducts. At present, a large number of exhaustive studies have focused on the biological synthesis of AgNPs using natural substances such as bacteria, fungi, proteins, polysaccharides, and plant extracts [5,13,14,15,16]. Considering the safety of biological synthesis process, the approaches involving plant extracts could generate much less contamination and have minimal impact on the environment. Besides that, the AgNPs synthesis using plant extracts which can act as both reducing and capping agents is also regarded as a cost-effective, time-saving, and easy operational process [17]. Moreover, the benefit of plant-mediated AgNPs synthesis also lies in the generation of large amounts of AgNPs with well-defined size and morphology. Recently, there is a growing trend towards the functionalization of silk through a coating process using biosynthesized AgNPs, which contributes to the expansion of the applications of silk materials.

**Figure 1:**
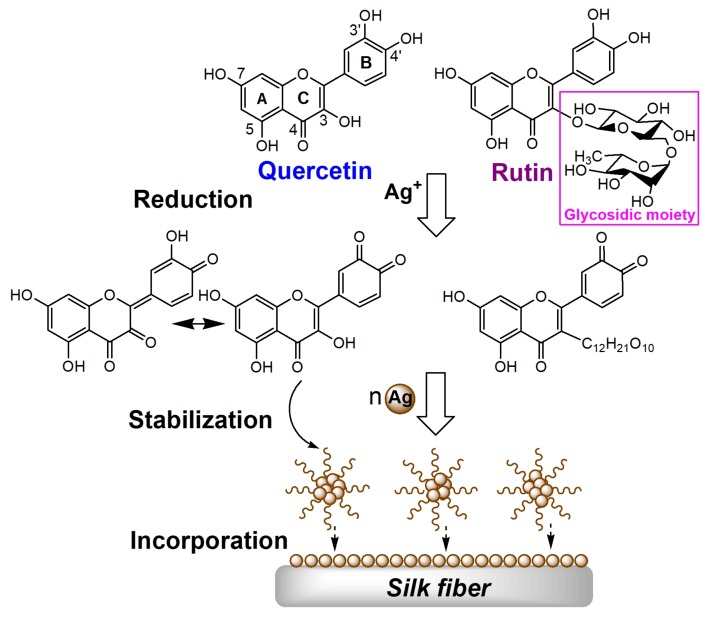
Scheme of the process of coating the AgNPs synthesized by flavonoids onto silk.

Flavonoids, as one dominant category in natural extracts, are widely distributed in plants forfulfilling many physiological functions [18]. Due to the advantages of flavonoids including biocompatibility, low toxicity, and eco-friendliness, their application to textiles for the coloration and functionalization purposes have been considered as a green and sustainable production process. Quercetin and rutin are two representative bioactive compounds in the family of flavonoids. The essential distinction between them is that there is a disaccharide moiety located at the C-3 position of C-ring in the chemical structure of rutin (Figure 1). Some previous reports have already studied the AgNPs synthesis using plant extracts containing flavonoids [19,20]. However, few reports are available for the preparation of the antibacterial silk textiles through the coating of AgNPs which are synthesized by the highly pure flavonoids with similar structures. Moreover, antioxidant activity is an important property of silk materials applied in medical and healthy clothing as well as bioactive dressings which can deactivate highly reactive and harmful species such as oxygen radicals. Our previous study has proved that quercetin- and rutin-treated silk showed excellent antioxidant activity [21], which enables us to further explore the antioxidant activity of the silk materials treated with the AgNPs prepared by quercetin and rutin. In addition, the functional silk fabrics are inevitably subjected to repeated washings during usage. Therefore, the durability of the antibacterial and antioxidant activities of the AgNPs-treated silk is also worthy of further study.

The coating process of the AgNPs prepared by two flavonoids on silk is schematically proposed in Figure 1. Generally, the mechanism of AgNPs synthesis in the presence of flavonoids lies in the diorthohydroxyl group in their B-ring which promotes the reduction of Ag^+^ into Ag^0^ by releasing two electrons. The flavonoids are eventually oxidized into the stable final product of 3′,4′-diquinone [22,23,24]. Both quercetin and rutin go through the aforementioned processes during the AgNPs synthesis. Moreover, for quercetin, the oxidation of hydroxyl groups can also take place in the 4- and 4′-positions [25]. As the reaction proceeds, Ag undergoes further aggregation to larger clusters and finally forms AgNPs. The quinone form of flavonoids is also attached to the surface of nanoparticles [26,27]. During the treatment process, the as-prepared AgNPs gradually approach to the silk surface and finally firmly deposit on silk. In addition, a certain amount of unoxidized flavonoids can also be adsorbed on silk in acidic conditions during the process [21].

The aim of this study was to fabricate bioactive silk fabrics by means of the coating by the AgNPs synthesized by two flavonoids. The synthesis factors including pH, flavonoid concentration and temperature were discussed. The stability of AgNPs colloids regarding the particle size and zeta potential over time was also studied. The morphology and crystalline structure of AgNPs were investigated using transmission electron microscopy (TEM) and X-ray diffraction (XRD), respectively. The surface morphology of the AgNPs treated silk was characterized by scanning electron microscope (SEM). The color characteristics of the silk fabrics treated with various concentrations of AgNPs were also measured. Finally, the antibacterial and antioxidant activities of the AgNPs coated silk as well as their durability were evaluated.

## 2. Materials and Methods

### 2.1. Materials

Crepe de chine silk fabric with specification code of 12,103 was obtained from Wujiang Zhiyuan Textile Co. Ltd., Suzhou, China. Quercetin (purity, 95%) and rutin (purity, 95%) were purchased from Xi’an Qing Yue Biotechnology Co. Ltd., Xi’an, China. All the chemicals used were analytical agents. A detergent especially designed for silk was used for the evaluation of washing durability. All water applied in this study was ultrapure water.

### 2.2. Synthesis of AgNPs

Stock solutions of flavonoids (2 mM) at pH 10 were freshly prepared. The AgNPs were synthesized by adding 5 mL stock solution to 45 mL AgNO_3_ solution (1 mM, final concentration). The mixture was then incubated in the oscillator at 50 °C shaking vigorously for a period of time. In the study of pH effect, the pH values were adjusted to 8, 9, and 10 by acetic acid and sodium carbonate. The effect of flavonoid concentration was investigated by changing the flavonoid concentration from 0.1 to 0.4 mM. To evaluate the temperature effect, the reaction mixtures were incubated at different temperatures (20, 40, 60, and 80 °C) for 60 min. The effect of reaction time was studied by incubating the reaction mixture for 0.5 to 100 min at constant temperature of 20 °C.

### 2.3. Preparation of AgNPs-Treated Silk

1 g silk fabric was immersed in the as-prepared AgNPs mixture (50 mL) at pH 4. The temperature was initially set at 30 °C, raised up to 90 °C at a speed of 2 °C/min, and kept at 90 °C for 60 min.

### 2.4. Characterizations of AgNPs

The absorbance and spectrum of the AgNPs solution were examined by the Shimadzu UV-1800 UV–Visible spectrophotometer (Shimadzu Co., Kyoto, Japan). Prior to the test, all the solutions were diluted 10 times in order to obtain the accurate UV–Vis absorption spectra. The particle size distribution and zeta potential of AgNPs were identified using the Zetasizer Nano ZS 90 (Malvern Instruments Ltd., Malvern, UK) based on the dynamic light scattering (DLS) analysis. The morphology of the AgNPs was observed through the HT7700 transmission electron microscope (Hitachi High Technologies America, Inc., Schaumburg, IL, USA). The average size and polydispersity of AgNPs were analyzed by the Nano Measurer 1.2.5 software (developed by Dr. Jie Xu in the Department of Chemistry at Fudan University, Shanghai, China) based on the TEM micrograph. The crystalline structure of AgNPs was detected by the X’Pert-Pro MPD diffractometer (PANalytical B.V., Utrecht, The Netherlands) using Cu-Kα radiation. The AgNPs prepared by 0.2 mM quercetin or rutin and 1 mM AgNO_3_ were used in the TEM and XRD measurements.

The cyclovoltammetry measurements were performed in a standard three-electrode system utilizing the PGSTAT302N electrochemical workstation (MetrohmAutolab B.V., Utrecht, The Netherlands). An Ag/AgCl, KCl sat electrode and a Pt rod were used as reference and counter electrode, respectively. The scan rate was 0.1 V/s. A citric acid/sodium hydrogen phosphate buffer solution (pH 5) was used as supporting electrolyte. According to the previous report [24], the flavonoids showed more obvious peak under acidic condition.

### 2.5. Characterization of AgNPs-Treated Silk

The surface morphology of silk fabric was observed by the Hitachi S-4800 SEM (Hitachi High-Technologies Co., Schaumburg, IL, USA). The a* (redness-greenness value) and b* (yellowness-blueness value) color parameters of silk fabrics were measured by Data color 600 (Datacolor Technology Co., Lawrenceville, NJ, USA) using D65 illumination with 10° observer; the fabrics were treated with different concentrations of flavonoids and AgNO_3_ (Table 1) following the process shown in the section of preparation of AgNPs treated silk.

The Ag content of silk fabrics was examined by the ICAP 6300 DUO (Thermo Fisher Scientific Inc., Waltham, MA, USA), and more descriptions were shown in our previous work [28]. The antibacterial activity of silk fabrics was determined according to our previously reported method [29,30]. The samples treated with the AgNPs synthesized by 0.2 mM quercetin or rutin and 1 mM AgNO_3_ were adopted. The antioxidant activity of silk fabrics was measured through the ABTS^+^ decolorization assessment by spectrophotometric analysis [31], and more details were present in our previous work [29,30]. The samples treated with the AgNPs synthesized by 0.2, 0.4, and 0.6 mM quercetin or rutin with 1 mM AgNO_3_ were used. The washing process of the AgNPs treated fabrics was undertaken in a washing solution containing 2 g/L detergent at 40 °C for 30 min, which was repeated 5, 10, and 30 times to get 5, 10, and 30 washing cycles; the silk fabrics treated with the AgNPs synthesized by 0.2 mM quercetin or rutin and 1 mM AgNO_3_ were used in this assessment.

## 3. Results and Discussion

### 3.1. Preparation of AgNPs

#### 3.1.1. Preparation Conditions

**Figure 2:**
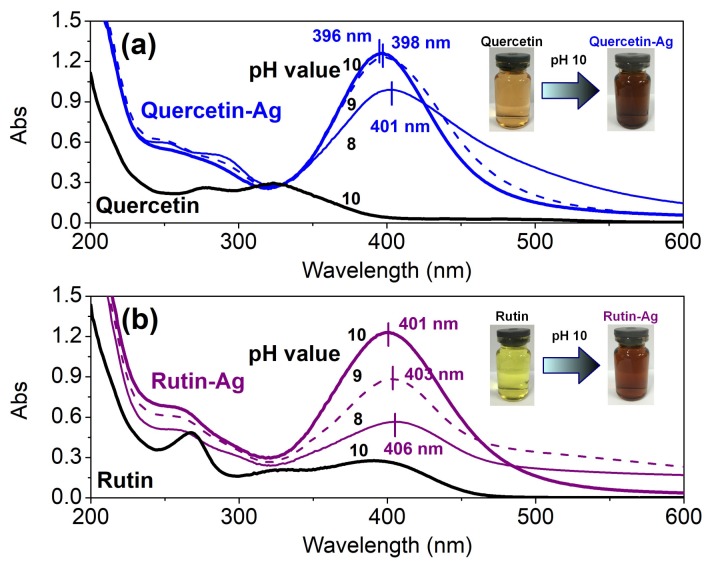
UV–Vis absorption spectra of the AgNPs synthesized by quercetin (**a**) and rutin (**b**) at various pHs.

*Effect of pH*: The pH value exerts a great impact on the physical and chemical properties of flavonoids [32] which are in close relationship with their solubility, dissociation degree, and reducing capability. Thus, in this work, 0.16 mM flavonoids and 1 mM AgNO_3_ were used for the AgNPs synthesis in the pH range of 8–10. As depicted in Figure 2 (black lines), quercetin and rutin showed two major bands in their UV–Vis absorption spectra. Band I in the 300–400 nm range and Band II in the 240–280 nm range represented the cinnamoyl system (B and C rings) and benzoyl system (A and C rings), respectively [33]. With the addition of AgNO_3_, the color of the solutions changed from faint yellow to brownish within few minutes, indicating the formation of AgNPs. This result also reveals the great reducing capability of quercetin and rutin. After continuous oscillating for 60 min, a series of clear and deep brownish solutions were obtained along with the appearance of intense peaks (λ_max_) at round 400 nm in their spectra (blue or purple lines). These optical phenomena called surface plasmon resonance are attributed to the cumulative oscillation of the conducting metal surface electrons in resonance with the non-particulate radiation [34]. Moreover, in both cases of quercetin and rutin, the absorbance at the maximum absorption wavelength increased gradually with increasing pH, suggesting the AgNPs are more inclined to form at higher pH due to the strengthened deprotonation of the phenolic groups [35]. In addition, the absorbance of the new peaks underwent a blue shift as the pH further increased due to the decrease of the mean size of AgNPs [36].

**Figure 3:**
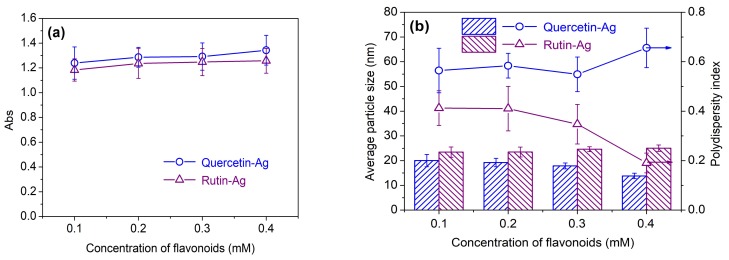
Absorbance (**a**) and average particle size (**b**) of the AgNPs synthesized using various concentrations of flavonoids.

*Effect of flavonoid concentration*: As depicted in Figure 3a, the absorbance of the AgNPs solution increased with increasing flavonoid concentration, indicating the rising yield of AgNPs. Moreover, the quantity of AgNPs produced by quercetin was higher than that of rutin at the same concentration, implying that quercetin has stronger reducing capability than rutin. Both quercetin and rutin were able to produce AgNPs with the particle size smaller than 25 nm (Figure 3b). As the concentration of quercetin increased, the average particle size of AgNPs showed a decrease tendency. This could be ascribed to the stabilizing effect of quercetin on AgNPs. However, there was no significant change in AgNPs particle size as the rutin concentration increased. This interesting phenomenon is related to the chemical structure of rutin. Because the DLS is sensitive to the size of the whole nanocomposites [37], the size ofAgNPs measured by DLS depends greatly on the coating layers on their surface.Rutin, as a stabilizer, can surround the surface of AgNPs, and its glycosidic moiety can form hydrogen bonds with other rutin or water molecules, thus making the diameter of the AgNPs larger at higher rutin concentration. From the results of the polydispersity index (PDI), it can also be seen that the PDI became lower as more rutin was used, indicating that the AgNPs were more uniformly distributed compared with the case of quercetin.

**Figure 4:**
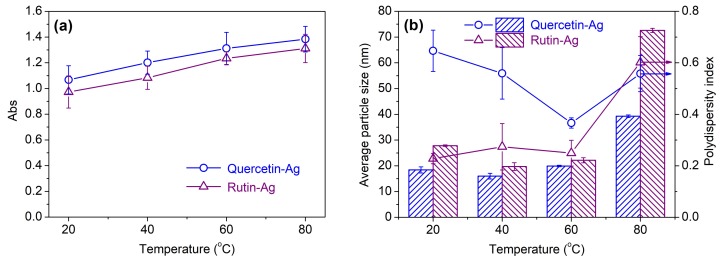
Absorbance (**a**) and average particle size (**b**) of the biosynthesized AgNPs at various temperatures.

*Effect of temperature*: The yield of AgNPs increased with the elevated temperature, as revealed by the increasing absorbance in Figure 4a. This result indicates that higher temperature can facilitate the production of more AgNPs, which is consistent with previous studies [35,38]. The temperature showed little impact on the particle size of AgNPs in the range of 20 to 60 °C, but exhibited an abrupt increase in particle size and PDI at 80 °C (Figure 4b). This phenomenon might be induced by the decreased stability of the AgNPs colloid at the high temperature which results in the agglomeration and poor uniformity of AgNPs. Considering the yield, particle size, and uniformity of AgNPs, 60 °C was used in the following experiments.

**Figure 5:**
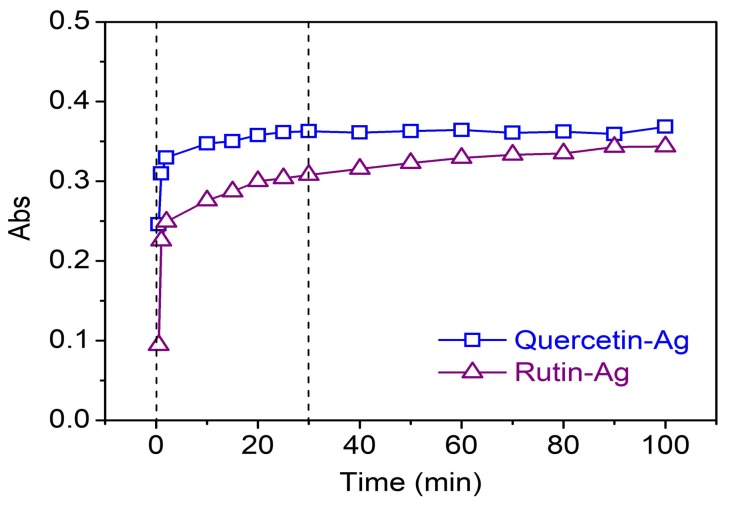
Absorbance of the biosynthesized AgNPs as a function of reaction time.

*Effect of time*: In order to study the generation of AgNPs in the presence of flavonoids against time, the absorbance of the AgNPs solution synthesized by 0.2 mM quercetin or rutin and 1 mM AgNO_3_ was measured as the time elapsed. The constant temperature was set at 20 °C due to the fast yield of AgNPs at high temperature which has an impact on the accuracy of results. As depicted in Figure 5, the concentration of AgNPs increased drastically in the first 30 min, and then gradually approached a constant with reaction time. No shifts in the peak wavelength were found during synthesis. More than 85%conversion was completed within 30 min, indicating the high efficiency of reduction reaction by flavonoids, which is beneficial to time and energy saving in practical applications. Moreover, the synthesis of AgNPs was faster by quercetin than by rutin, revealing that quercetin possesses higher reducing capability than rutin, which is further confirmed by the cycle voltammetry study.

The above experiments demonstrate the successful preparation of AgNPs. The present synthesis of AgNPs using quercetin and rutin possesses the following advantages: (a) non-toxic flavonoids from raw plants are used; (b) flavonoids perform the dual role of reducing and stabilizing agents and the need to employ additional stabilizing agents is eliminated; (c) the application of high pure flavonoids can avoid the presence of the useless chemicals in AgNPs solutions, which is beneficial to obtain clean and functional silk by treatment of the as-prepared AgNPs solutions; (d) water is used as a non-toxic solvent, and the resulting AgNPs can be directly applied to the treatment of silk; (e) AgNPs can be rapidly synthesized in mild conditions, indicating low energy consumption. These benefits conform to the trend of green chemistry and the demand of sustainable development [39].

#### 3.1.2. Cycle Voltammetry Study

In this section, the electrochemical oxidation of quercetin and rutin were investigated by cycle voltammetry in order to further compare their reducing capabilities. As depicted in Figure S1, the anodic potentials of quercetin and rutin were 0.208 and 0.241 V, respectively. This result reveals that quercetin has a higher reducing capacity than rutin, which is also consistent with the previous studies [25,40].

#### 3.1.3. TEM and Particle Size Distribution

**Figure 6:**
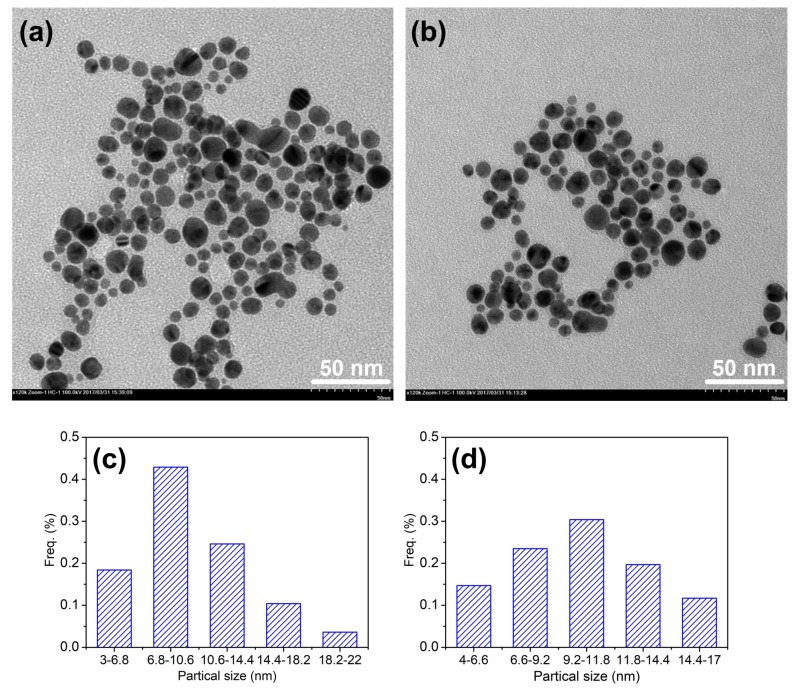
TEM images and their corresponding particle size distribution histograms for AgNPs prepared by quercetin (**a**,**c**) and rutin (**b**,**d**).

The TEM is a significant testing method for further investigating the morphology and size distribution of the AgNPs prepared by flavonoids. As seen in Figure 6a,b, the majority of the AgNPs were spherical in shape and well monodispersed. The particle size distribution of the AgNPs synthesized by quercetin and rutin (Figure 6c,d) was 3–22 and 4–17 nm, respectively. The results were much smaller than those measured by the DLS method (Figure 3 and Figure 4). Similar phenomena were also observed in our previous studies [28,38]. It is because that the TEM is only to detect the electron-rich metal particles, but the DLS analysis presents the result of the size of the whole nanocomposites [37]. Furthermore, three major factors are responsible for the size differences between two methods: the presence of flavonoids on the AgNPs surface, the aggregation of some small particles on the stabilized AgNPs, and the adsorption of water on the stabilized AgNPs [38,41]. In this regard, the adsorption of flavonoids on the surface of AgNPs contributes the most. In other words, the larger size of AgNPs measured by the DLS method than that by the TEM analysis also reveals that the AgNPs are coated by flavonoids as stabilizers. Moreover, it can also be seen that the particle size of AgNPs synthesized by quercetin was smaller but showed larger distribution range than that of AgNPs synthesized by rutin, which is in agreement with the results obtained by the DLS method.

#### 3.1.4. XRD Analysis

As seen in Figure S2, the XRD patterns confirmed the successful synthesis of AgNPs by flavonoids, which showed intense peaks located at 38.3°, 44°, 64.6°, and 77.4°, respectively representing (111), (200), (220), and (311) Braggs reflections of face centered cubic structure of Ag [42]. This result illustrates that the as-prepared AgNPs are crystalline in nature. Those XRD patterns are also in line with the standard JCPDS file No. 04-0783.

#### 3.1.5. Stability of AgNPs

**Figure 7:**
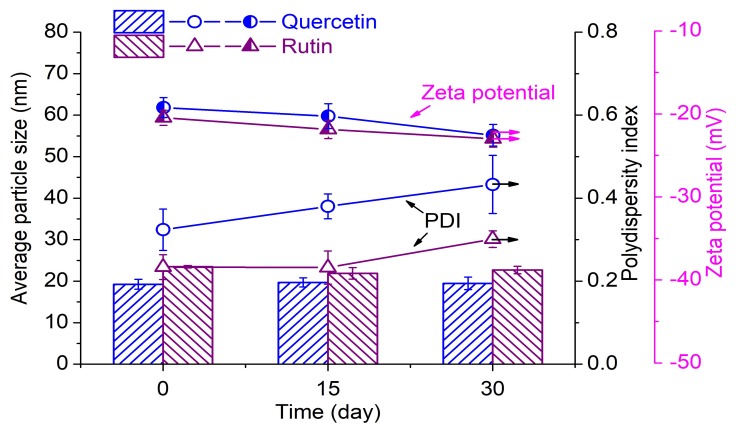
Changes in the particle size, polydispersity index, and zeta potential of AgNPs as a function of time.

The good stability of AgNPs is of importance during their application. Thus, the UV–Vis absorption spectrum, average particle size, and zeta potential of AgNPs as a function of storing time were investigated. The AgNPs solutions prepared by 0.2 mM flavonoids and 1 mM AgNO_3_ at pH 9 were stored in the sealed flask at room temperature (25 °C) for measurements. As depicted in Figure S3, the absorbance of AgNPs solutions gradually increased along with the time, which is caused by further reaction in the solution. There was almost no change in the peak shape of the AgNPs solution, indicating that no agglomeration of AgNPs or generation of new organic substance took place within 30 days. It can be seen from Figure 7 that the AgNPs prepared by quercetin and rutin showed good stability in particle size. The PDI of the AgNPs solution showed a slight increase after 30 days. The AgNPs synthesized by rutin displayed a lower PDI than those synthesized by quercetin. This is attributed to the glycosidic moiety present in the rutin molecule which can contribute to a better stability of AgNPs. The zeta potentials of the AgNPs mixture prepared by quercetin and rutin were −19.2 and −20.5 mV, respectively, indicating that the surfaces of the AgNPs colloids were negatively charged. This phenomenon is due to the quinone form of flavonoids with several hydrolyzed hydroxyl groups attached to the surface of nanoparticles, which can avoid the agglomeration and sedimentation due to the electrostatic repulsion between the particles (Figure 1) [26,27]. In addition, the AgNPs solution showed an increase in the absolute value of zeta potential during storage, revealing the enhancement of stability. In conclusion, the AgNPs solutions prepared by flavonoids have good stability with no sign of aggregation in 30 days.

### 3.2. Application of AgNPs

#### 3.2.1. Color Characteristics

**Figure 8:**
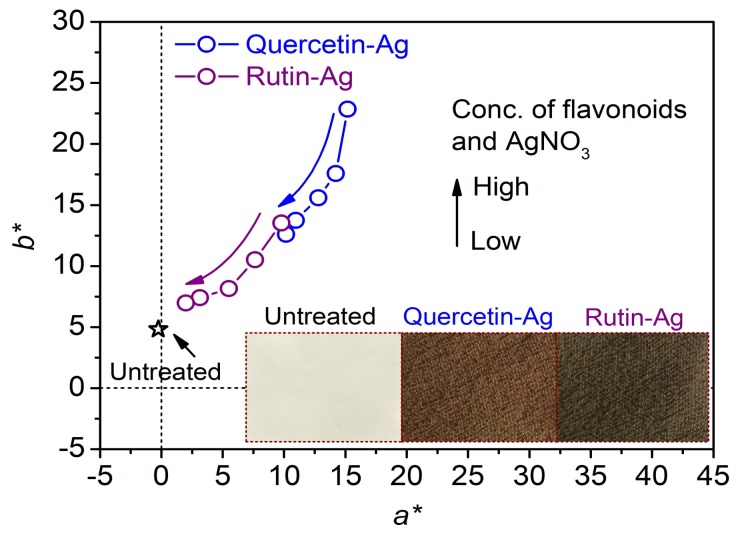
Color parameters of the silk fabrics treated with the AgNPs synthesized with different concentrations of flavonoids and AgNO_3_.

Figure 8 shows the a*/b* values of the AgNPs treated silk fabrics measured according to the CIE color system, and the photographs of the silk treated with the AgNPs synthesized by 0.2 mM flavonoids and 1 mM AgNO_3_. The original silk fabric had a colorless and shinning appearance, and its a*/b* point was close to the origin in the color space. The silk fabrics treated with quercetin and rutin also displayed a faint yellow color [43]. After treated with the AgNPs, the silk fabrics exhibited a brownish color. Moreover, as the concentration of AgNPs increased, the a*/b* plots of the treated silk fabrics shifted towards the origin of coordinate, implying the decreased color saturation and the dull color. From Figure 8, it can also be concluded that the color characteristics of the AgNPs-treated silk fabrics are related to the concentration of AgNPs and the species of flavonoids used for the AgNPs synthesis.

#### 3.2.2. Surface Morphology

**Figure 9:**
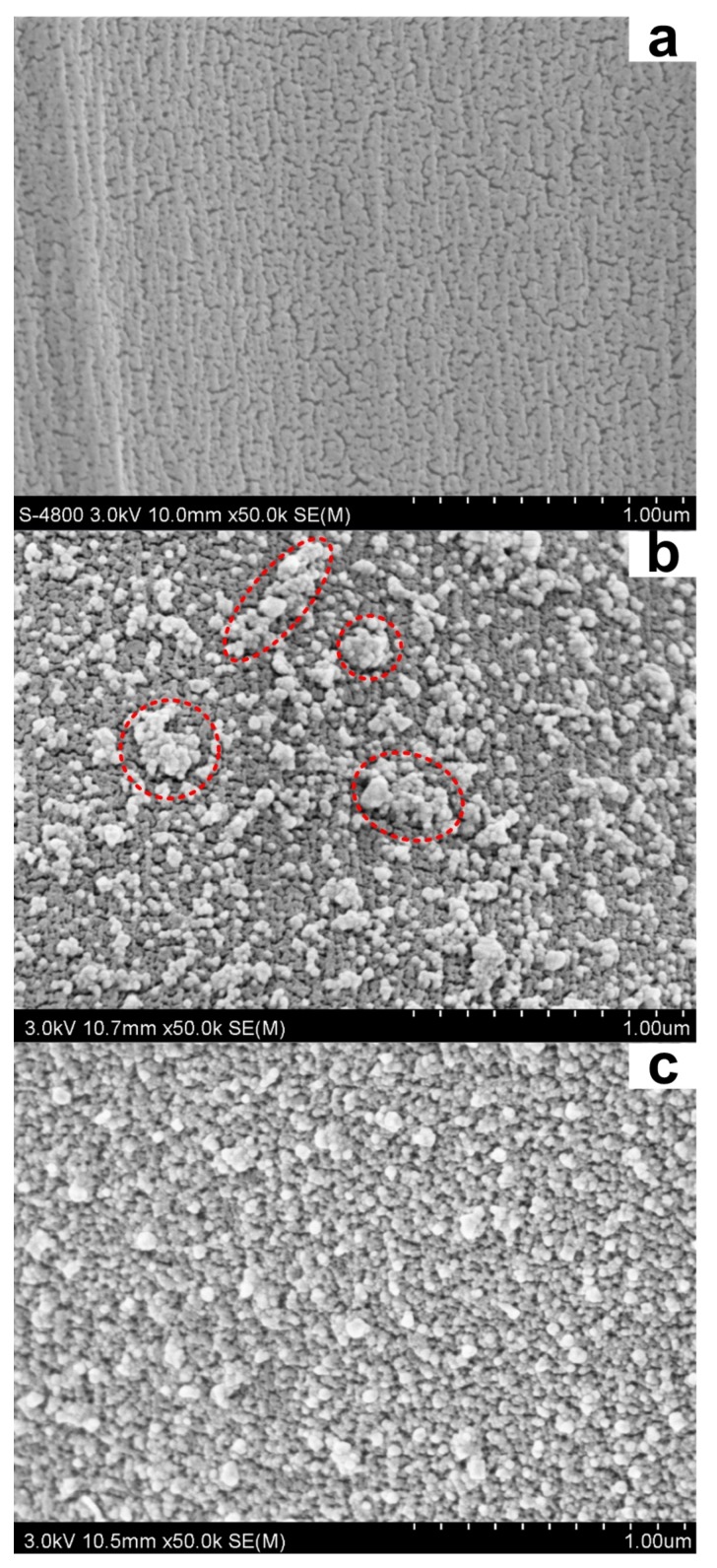
SEM images of the untreated silk (**a**) and the silk treated with AgNPs synthesized using quercetin (**b**) and rutin (**c**).

Figure 9 shows the surface morphologies of the silk treated with the AgNPs prepared by 0.2 mM flavonoids and 1 mM AgNO_3_. As shown in Figure 9a, the original silk fiber has a smooth and clean surface. However, large quantities of spherical-shaped AgNPs were uniformly distributed on the surface of the treated silk (Figure 9b,c). Moreover, the majority of AgNPs on silk surface had a diameter around 50 nm which was larger than those of the AgNPs colloid measured by DLS and TEM. This phenomenon is caused by the high surface energy of AgNPs on the silk surface, leading to the aggregation of AgNPs [44]. In addition, the AgNPs prepared by rutin were found to display better distribution uniformity on silk than those synthesized by quercetin. This may be attributed to the fact that the rutin prepared AgNPs exhibit better dispersion performance in solution induced by its glycosidic moiety than the quercetin prepared AgNPs, facilitating a lower adsorption rate during the coating process. In brief, the SEM images strongly confirm the successful coating of AgNPs onto the silk surface. The high density and uniform dispersity of AgNPs on silk is essential for the antibacterial activity.

#### 3.2.3. Antibacterial and Antioxidant Activities

**Figure 10:**
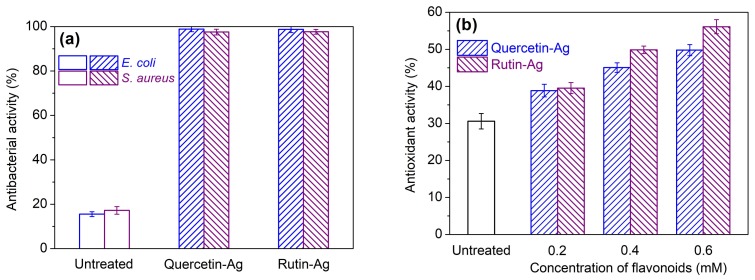
Antibacterial (**a**) and antioxidant (**b**) activities of the AgNPs-treated silk fabrics.

The antibacterial activity against *E. coli* (Gram−) and *S. aureus* (Gram+) of the AgNPs treated silk was evaluated. As seen in Figure 10a, the original silk had very poor antibacterial activity, and its bacterial reduction rate against *E. coli* and *S. aureus* was 16% and 17%, respectively. The AgNPs-treated silk displayed excellent antibacterial activity. In other words, the silk treated with the AgNPs prepared by quercetin or rutin inhibited over 97% of *E. coli* and *S. aureus* in 24 h. The excellent antibacterial activity of the AgNPs treated silk stems from the AgNPs which can attach to the bacterial cell membrane and damage its sulfur-containing proteins or even penetrate into the bacteria and interact with the phosphorus containing compounds like DNA [45]. Moreover, the Ag^+^ ions released from AgNPs also make contributions to the enhancement of antibacterial activity [46]. From Figure 10a, it was also found that the bacterial reduction against *E. coli* was slightly higher than that against *S. aureus*. This can be explained by the different thicknesses of the cell wall of Gram-positive and Gram-negative bacteria [43,47]. In addition to the contribution of AgNPs to antibacterial activity, a certain amount of quercetin and rutin adsorbed by silk during the treatment can also participate in the bacterial reduction.

As seen in Figure 10b, the untreated silk had a low antioxidant activity of 30%. After treated with AgNPs prepared by 0.2 mM quercetin and rutin, the silk fabrics showed antioxidant activity of 39% and 40%, respectively. This result indicates that the AgNPs prepared by flavonoids can improve the antioxidant activity of silk. However, the silk samples treated with the flavonoid prepared AgNPs displayed much lower antioxidant activity than those directly treated with the equivalent concentration of flavonoids according to our previous work [21]. This is due to the fact that the phenolic hydroxyl groups of catechol moiety in the B ring of the flavonoids are mainly responsible for radical scavenging [48], but they are oxidized and converted into the quinine form during the AgNPs synthesis. In addition, the free hydroxyl groups present in the A ring of flavonoids also possess a certain degree of free radical scavenger potential [49], contributing to the antioxidant activity of the treated silk. Furthermore, the low adsorption quantity of flavonoids on silk is another reason for the poor antioxidant activity of the treated silk, due to the high pH and low temperature used during the treatment process [21] as well as the hindrance of the AgNPs coating layer. Though all of the above-mentioned discussions, increasing the concentration of flavonoids during AgNPs synthesis was able to enhance the antioxidant activity of the treated silk fabric, as shown in Figure 10b.

#### 3.2.4. Durability of Functionalities to Washing

**Figure 11:**
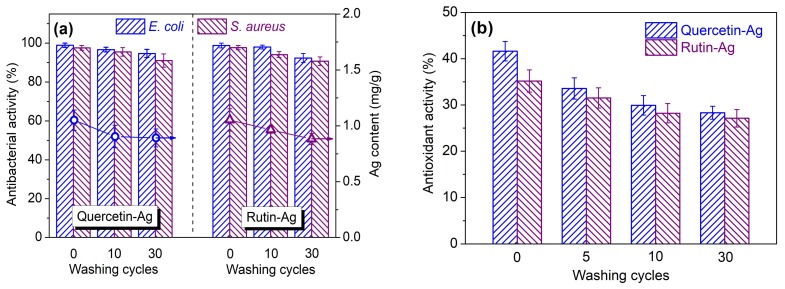
Washing durability of the antibacterial (**a**) and antioxidant (**b**) activities of the silk fabrics treated with AgNPs synthesized using quercetin and rutin.

Textiles are inevitably subjected to constant washings during usage. Textiles with good washing durability can prolong their life-span. In this work, the durability of the antibacterial and antioxidant activities of the AgNPs treated silk fabrics towards repeated washing cycles was evaluated. As depicted in Figure 11a, the antibacterial activity of the AgNPs treated silk gradually decreased with increasing washing cycles. After 30 repeated washing cycles, the antibacterial activity of the treated silk still remained over 90% against both strains. This result manifests that the AgNPs are firmly fixed on the surface of silk and only a small amount of AgNPs is released from the fiber as confirmed by the Ag content analysis. The antioxidant activity of the treated silk showed obvious decline for the initial washing, and then decreased slowly until the last round of washing (Figure 11b). This indicates that the partially oxidized flavonoids have a low affinity to silk fiber, and they are released gradually from silk during washing. In conclusion, the silk fabrics treated with the flavonoid-prepared AgNPs can retain long-term antibacterial activity and provide a continuous supply of antioxidant activity for sustained-release purposes.

## 4. Conclusions

This study presents a facile and eco-friendly approach towards the preparation of the AgNPs coated silk materials using quercetin and rutin. The synthesis factors including pH, temperature and flavonoid concentration on the yield and particle size of AgNPs were discussed. The color characteristics and bioactivities of the treated silk were also studied. The results showed that more AgNPs were produced at higher pH values. The optimum temperature for the AgNPs synthesis by quercetin and rutin was 60 °C in view of the yield, particle size, and uniformity of AgNPs. Quercetin showed higher reduction capability than rutin. The AgNPs colloids prepared by flavonoids remained stable after 30 days. The AgNPs prepared by rutin were distributed more evenly on silk than those synthesized by quercetin. The AgNPs-treated silk fabrics displayed bacterial inhibition rates over 90% against *E. coli* and *S. aureus* even after 30 washing cycles and gradual decrease in antioxidant activity during washing.

## Figures and Tables

**Table 1:** Concentrations of flavonoids and AgNO_3_.

Flavonoids (mM)	AgNO_3_ (mM)
0.10	0.50
0.15	0.75
0.20	1.00
0.25	1.25
0.30	1.50

## Supplementary Materials

The following are available online at http://www.mdpi.com/2073-4360/10/4/404/s1, Figure S1: Cyclic voltammograms of flavonoids, Figure S2: XRD patterns of the AgNPs synthesized using flavonoids, Figure S3: Variations of the UV–Vis absorption spectra of the AgNPs solutions as a function of time.
