# Supplementary material for: Facile and Eco-Friendly Fabrication of Colored and Bioactive Silk Materials Using Silver Nanoparticles Synthesized by Two Flavonoids
Source: Polymers (Basel). 2018 Apr 4;10(4):404. doi: 10.3390/polym10040404 (PMC6415457; doi:10.3390/polym10040404)
Supplement: Supplementary file 1 [file polymers-10-00404-s001.pdf]

Supplementary Materials

Polymers

Facile and eco-friendly fabrication of colored and bioactive silk materials using silver nanoparticles synthesized by two flavonoids

Yuyang Zhou [1] and Ren-Cheng Tang [1,*]

[1] National Engineering Laboratory for Modern Silk, College of Textile and Clothing Engineering, Soochow University, 199 Renai Road, Suzhou 215123, China; ldxyzyy@163.com (Y.Z.)

* Correspondence: tangrencheng@suda.edu.cn (R.-C.T.); Tel.: +86 512 6716 4993

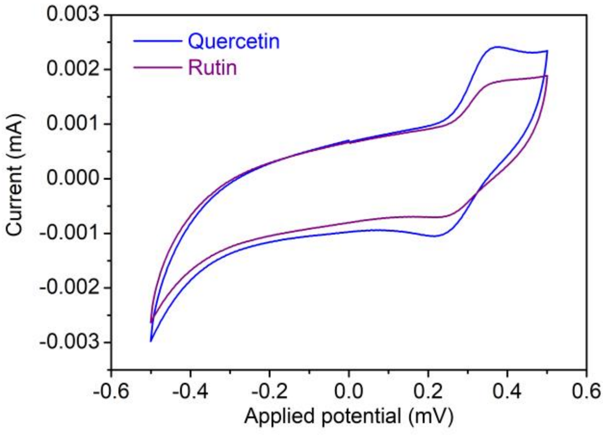

Figure S1. Cyclic voltammograms of flavonoids.

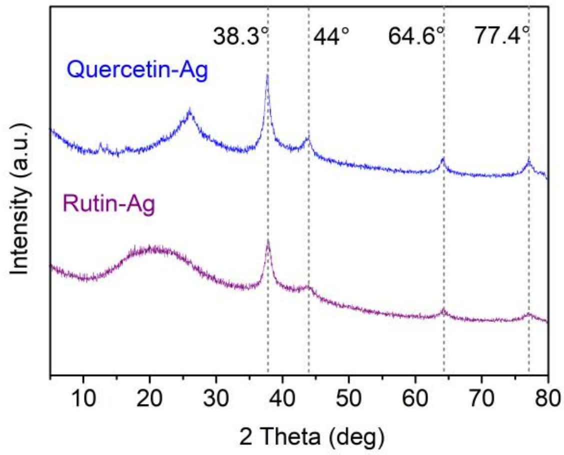

Figure S2. XRD patterns of the AgNPs synthesized using flavonoids.

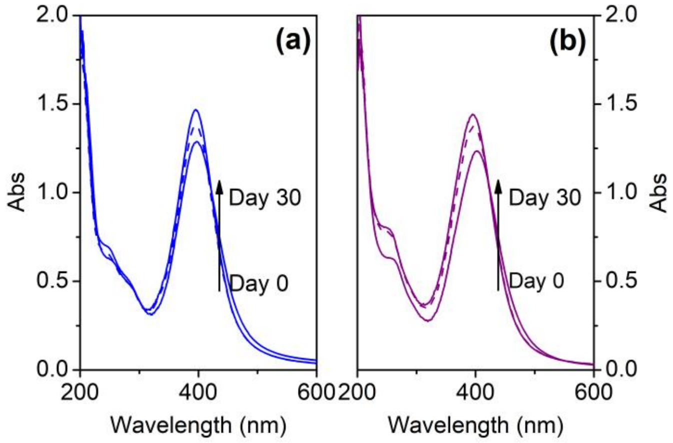

Figure S3. Variations of the UV-Vis absorption spectra of the AgNPs solutions as a function of time.
